# Fluorescence in situ hybridization in combination with the comet assay and micronucleus test in genetic toxicology

**DOI:** 10.1186/1755-8166-3-17

**Published:** 2010-09-15

**Authors:** Galina G Hovhannisyan

**Affiliations:** 1 Department of Genetics and Cytology, State University, Biological Faculty, 1 Alex Manoukian Street, Yerevan 375025, Armenia

## Abstract

Comet assay and micronucleus (MN) test are widely applied in genotoxicity testing and biomonitoring. While comet assay permits to measure direct DNA-strand breaking capacity of a tested agent MN test allows estimating the induced amount of chromosome and/or genome mutations. The potential of these two methods can be enhanced by the combination with fluorescence *in situ *hybridization (FISH) techniques. FISH plus comet assay allows the recognition of targets of DNA damage and repairing directly. FISH combined with MN test is able to characterize the occurrence of different chromosomes in MN and to identify potential chromosomal targets of mutagenic substances. Thus, combination of FISH with the comet assay or MN test proved to be promising techniques for evaluation of the distribution of DNA and chromosome damage in the entire genome of individual cells. FISH technique also permits to study comet and MN formation, necessary for correct application of these methods. This paper reviews the relevant literature on advantages and limitations of Comet-FISH and MN-FISH assays application in genetic toxicology.

## Introduction

A considerable battery of assays exists for the detection of different genotoxic effects of compounds in experimental systems *in vitro*, or for investigations of exposure to genotoxic agents *in vivo*. The single cell gel electrophoresis, called shortly 'comet assay', as well as the micronucleus (MN) test are broadly applied test systems to check for genotoxic effects. In addition to classical cytogenetic methods for scoring chromosomal aberrations, fluorescence *in situ *hybridization (FISH) is used in genetic toxicology for analysis of chromosome damage with increased efficiency and specificity for identifying certain kinds of chromosomal aberrations. The comet assay, MN test and FISH presented in International Programme on Chemical Safety (IPCS) guidelines among the most often studied genotoxicity endpoints for the monitoring of genotoxic effects of carcinogens in humans [1]. Recently FISH technique was successfully combined with comet and MN assays for simultaneously measuring the overall level of DNA and chromosome damage, and localizing of specific genome domains within an individual cell.

### Principles and application of the comet assay

The comet assay is a rapid and very sensitive fluorescent microscopy-based method for measuring DNA damage, protection and repair at the level of individual cells [2-7]. In this assay cells are embedded in agarose, lysed and then electrophoresed. Negatively charged broken DNA strands exit from the lysed cell under the electric field and form a comet with "head" and "tail." The amount of DNA in the tail, relative to the head, is proportional to the amount of strand breaks. The limit of the comet assay sensitivity is approximately 50 strand breaks per diploid mammalian cell [8]. It permits to reveal mainly early, still repairable, moderate DNA damage and can be used in virtually any eukaryotic cell. In order to achieve various objectives, different modifications of the comet assay have been developed. In its alkaline version, which is mainly used, DNA single-strand breaks, DNA double-strand breaks, alkali-labile sites, and single-strand breaks associated with incomplete excision repair sites cause increased DNA migration [9]. In the neutral variant the DNA molecule itself is preserved as a double stranded structure which enables uncovering of double stranded DNA breaks [10,11]. Crosslinkage of DNA-DNA/DNA-protein leading to decreased DNA migration can be identified by the failure to detect single-strand breaks that were known to be present [12]. Oxidized purins and pyrimidins, could be revealed by incubating lysed cells with base damage-specific endonucleases before electrophoresis [13]. The comet assay has manifold applications in fundamental research for DNA damage and repair, in genotoxicity testing, human biomonitoring and molecular epidemiology and ecotoxicology [5,14,15].

### Principles and application of MN test

The MN test is one of the preferred methods for assessing DNA damage at the chromosome level. It permits to measure both chromosome loss and chromosome breakage [16,17]. Metaphase analysis provides the most detailed analysis of numerical and structural chromosome aberrations, however, it is very time consuming and needs highly skilled personnel. The MN assay was developed as a simpler short-term screening test and now accepted as valid alternative to the chromosome aberration assay. In this method, chromosome aberrations are detected indirectly via chromatin loss from the nucleus leading to MN in the cytoplasm of the cell [18,19]. MN are expressed only in dividing cells. Adding to cell cultures cytochalasin-B, an inhibitor of the mitotic spindle that prevents cytokinesis, permits to recognize cells that have completed one nuclear division by their binucleated appearance [20,21]. The cytokinesis-block micronucleus (CBMN) assay allows higher precision because the data obtained are not affected by altered cell division kinetics [22]. Recently the CBMN assay has in fact evolved into a "cytome" method for measuring chromosomal instability, DNA repair capacity, nuclear division rate, mitogenic response and occurrence of necrotic and apoptotic cells [23]. The MN test has become one of the most commonly used methods in genotoxicity testing and biomonitoring populations at risk [15,24,25]. This test has been recommended for monitoring in product development and regulatory tests of new drugs [26].

### Principles and application of FISH technique

FISH is a powerful technique for localization of specific DNA sequences within interphase chromatin and metaphase chromosomes and the identification of both structural and numerical chromosome changes. The detection of nucleotidic sequences on examined DNA molecule consists in hybridizing a DNA probe to its complementary sequence on chromosomal preparations. Probes are labeled either directly, by incorporation of fluorescent nucleotides, or indirectly, by incorporation of reporter molecules that are subsequently detected by fluorescent antibodies. Probes and targets are finally visualized *in situ *by microscopy analysis. FISH technique protocols and wide variety of current applications of FISH technology are presented [27-33]. Structural and numerical chromosomal aberrations have been considered important biological end points in genotoxic studies. FISH with chromosome-specific DNA probes has increased the sensitivity and ease of detecting chromosomal aberrations, especially stable chromosomal aberrations. Now FISH is being increasingly utilized in genetic toxicology for the detection of chromosome damage induced *in vitro *and *in vivo *by chemical and physical agents [34-37].

### Overcoming of limitations of comet and MN assays by FISH

Compared with other assays, analysis of comets and MN bring along several advantages, including speed and ease of analysis and no requirement for metaphase cells in MN test and no need for dividing cells in the comet assay. However, results from the comet assay alone reflect only the level of overall DNA damage in single cells. The same is typical for MN test as it does not even permit to distinguish MN containing whole chromosomes from MN containing chromosome fragments. The introduction of FISH [27] in comet and MN assays has allowed adding new abilities and to enhance resolution and validity of these two methods.

FISH permitted to supplement potential of the comet assay with an opportunity to recognize genome regions of interest on comet images. Thus, Comet-FISH is applied for the analysis of damage and repair of different genes, chromosomes and chromosome regions compared to whole genomic DNA within the comet, or visualization of genomic loci in three-dimensional organization of chromatin and elucidation of mechanism of comet formation and DNA organization in comets. By MN test combined with FISH the genetic contents of the MN can be characterized. The application of FISH probes allows to distinguish MN originating either from chromosome loss or breakage and to determine the involvement of specific chromosomes and chromosome fragments in MN formation. Using MN-FISH the clastogenic or aneugenic action of different factors, the chromosomal origin of spontaneous and mutagen-induced MN, and the relative contribution of all chromosomes in MN formation can be studied.

Therefore, FISH was recognized as a valuable addition to comet and MN assays [38,39]. The simultaneous use of these methodologies will enable to achieve a higher sensitivity for the adequate hazard assessment of mutagens and will lead to a better understanding of the biological mechanisms involved. Literature data concerning combined application of FISH with comet and MN assays in genetic toxicology are discussed in the following.

## Comet-FISH

### Methodological aspects of Comet-FISH

Comet-FISH was first applied in human cells to compare the localization of specific chromosomal domains in native interphase nuclei with their distribution in comet-head and -tail after electrophoreses [40]. As heat-denaturation necessary for FISH is impossible within a comet fixed in low melting point agarose chemical denaturation of the DNA with alkali solutions was introduced [40] and applied in human leukocytes and the cell line HT1376 [41]. Soon thereafter the term ''Comet-FISH'' was introduced [42]. Two versions of Comet-FISH, one based on the alkaline and one on the neutral versions of the comet assay were developed subsequently [38,43]. The reliability of Comet-FISH was confirmed in some experiments. It could be shown that in Comet-FISH comparable results to metaphase based molecular cytogenetic approaches are obtained with respect to hybridization sensitivity and reproducibility [44] and the proportion of DNA elements from specific chromosomal domains in comet heads and tails corresponded to the expected localization based on the distribution of cleavage sites for specific endonucleases [45].

Various DNA probes were successfully applied with the comet assay for analysis of damage and repair of specific genome loci (genes, chromosomes and chromosome regions). The size of the region of interest investigated by Comet-FISH varies from gene [46] to whole chromosome [44]. In the comet assay 10 to 800 kb fragments are analyzed and fragments smaller than 10 kb might get lost in agarose gel [44]. However, DNA probes smaller than 10 kb cannot be used also in routine FISH. Results of various FISH probes application with the comet assay is summarized in Table 1.

**Table 1:** Results of various FISH probes application with the comet assay

FISH probes	What is detected	Results and applications	References
-	Whole DNA damage and repair	Genotoxicity testing or biomonitoring of genotoxic exposure and effect	[1-15]

Gene-specific	DNA damage and repair within the vicinity of the gene of interest	Analysis of damage and repair of genes related with cancer (*TP53, KRAS, APC, p53*)	[41,46,51,53-58].
		
		Analysis of damage of genes *Ret, Ab1 *and *Trp53 *as biomarker of X-radiation exposure *in vivo*	[59]

		Spatial distribution of chromosome-specific DNA sequences	[40]
		
Chromosome locus-specific (centromere-, telomere- and region-specific)	DNA damage within the vicinity of the locus of interest	Analysis of leukaemia-specific chromosome damage	[61]
		
		Analysis of sensitivity of telomeres toward anticancer drugs	[63-65]

WCP	DNA damage within the chromosome of interest	Distribution of DNA damage in genome	[44,60]
		
		Genetic alterations in carcinogenesis of the upper aerodigestive tract	[62]

Selected probes	Different genomic regions	Transcription-coupled DNA repair	[48]
		
		Elucidation of comet formation	[44-46,51,52]

Microscopic evaluation of the Comet-FISH images includes record of the number of probe signals and their localization on comet. The position of the fluorescence signals indicates whether the sequence of interest lies within the undamaged (head) or within or in the vicinity of a damaged (tail) region of DNA. Repositioning of gene-specific signals from tail to head over the incubation period provides evidence for repair of all the lesions within and around the locus of interest [47]. The level of DNA-damage and -repair in specific domains can be expressed as percent of FISH signals present in head vs. tail [48].

However, doing FISH on comet assay preparations is different from that of routine FISH mainly by the fact that it is performed not on flattened interphase nuclei fixed to a glass slide but on three-dimensional (3-D) preserved ones. This 3-D state reflects to some degree the real organization of chromatin in the living cell. On the other hand, the 3-D shape leads also to serious difficulties in the visualization and scoring of the signals [47]. Thus, analysis of Comet-FISH images is not easy automatized as the individual analysis of each comet is necessary to determine the distribution of signals between head and tail. Nevertheless, it is expected that automated systems for scoring of at least certain kinds of FISH signals might be elaborated in the near future [47].

### FISH in elucidation of comets formation

Although the comet method is very popular, there is still no agreement on how the comet tail is formed. Understanding of comet formation and the factors influencing this process is necessary for the comet assay correct application in genetic toxicology. In the comet assay, cells are electrophoresed in a way that fragmented and relaxed DNA migrates towards anode further than intact DNA, producing a situation resembling a comet. Relaxation of DNA loops was proposed to be the primary basis for comet formation under neutral [11,49] and alkaline [4] conditions. The comet tails obtained after neutral electrophoresis seem to consist of DNA loops which are attached to structures in the nucleus, since the DNA cannot move in the second direction after two-dimensional electrophoresis. Under alkaline electrophoresis conditions, however, the entire comet tail moves in the new electrophoresis direction. Thus, it appears that the alkaline comet tails consist of free DNA fragments [50].

The application of FISH has allowed to explain further aspects of comet formation. An important question is whether the complementary strands within a loop migrate into the tail independently or together upon alkaline denaturation and electrophoresis. Comet-FISH with a probe for the *p53 *gene was applied to cells that had been damaged by ionizing radiation. The results obtained favored the idea that both strands in a loop migrate into the tail, but separately, even in cases in which one strand is broken and the complementary strand is intact [51].

Before the era of Comet-FISH it was generally accepted that, when single cell gel electrophoreses is performed undamaged DNA remains in the comet head and the fraction of damaged DNA moves to the comet tail. FISH experiments indicated that besides the presence of breaks there are other factors determining the ability of particular DNA-sequences to migrate into the tail. These include the nature of the damage and organization of chromatin [47]. DNA from regions closely and extensively associated with the nuclear matrix, such as replicating DNA, does not move into the tail in standard alkaline comet assay [52]. Furthermore, fragments of gene-poor chromosomes were found more frequently in comet tail of UV-A-irradiated lymphocytes than fragments of gene-rich ones. It was suggested that chromosomes with high gene density are more resistant to DNA-damaging agents [44]. An alternative explanation would be the association of gene-rich regions with sites of transcription, which are located on the surface of the nuclear matrix i.e. in the head of the comet [44]. Similarly, the inability of the *DHFR *gene and one of ends of *MGMT *gene to leave the comet head even when the DNA is released from its supercoiling in CHO cells was explained by their attachment to 'matrix-associated region' [46]. Thus, the data obtained with Comet-FISH contributed in understanding of comet formation and correct interpretation of the comet assay results.

### Comet-FISH applications in genetic toxicology

Combined application of FISH with the comet assay offered the unique possibility to evaluate gene or chromosome damage and repair relative to the overall genome and compare repair rates of individual genes This methodology permits to detect mutagen-induced site-specific breaks in DNA regions that are relevant for development of various diseases or to recognize genome targets of action of environmental genotoxic agents. Recognition of sites of damage, promotes interpretation of induced *in vitro *and *in vivo *genotoxic effects and understanding of their biological impact.

#### FISH in study of gene damage and repair

Comet-FISH was found to be suitable for detection of DNA damage induced by genotoxic compounds e.g. in colon cancer relevant genes (*TP53, KRAS, APC*) in primary human colon and colon adenoma LT97 cells. Here this approach really facilitated studies on effects of nutrition-related carcinogens [53-56].

Comet-FISH revealed that strand breaks in the human tumor suppressor *p53 *gene are repaired very quickly compared with total DNA in RT4 and RT112 bladder carcinoma cells after γ-irradiation [57] and in mitomycin C-treated RT4 cells [51]. Preferential repair of the *p53 *locus was shown also in the panel of malignant breast cancer cell lines (MCF-7, MDA-MB-468 and CRL-2336) [58] and in normal lymphocytes [46] following genotoxic treatment.

Comet-FISH is also an effective alternative for measuring transcription-coupled repair (TCR), since the comet assay constitutes an extremely sensitive test for detection of DNA damage and repair by genotoxic agents at subtoxic, physiologically relevant exposures. Application of Comet-FISH for analysis of TCR was discussed elsewhere [48].

Localization and repair rates of *DHFR *and *MGMT *genes in CHO cells and *p53 *gene in human cells treated with H_2_O_2 _or photosensitizer plus light to induce oxidative damage were monitored using Comet-FISH with oligonucleotide probes for 5' and 3' regions of the genes investigated. CHO cells shown preferential repair of oxidative damage in the *MGMT *gene. Strand breaks in the human *p53 *gene were repaired more rapidly than total DNA. This approach can be applied to other genes treated with a range of damaging agents [46].

It has been shown, that damage of specific genes can be applied as biomarkers of genotoxic exposure. *Ret, Ab1 *and *Trp53 *genes fragmentation in Comet-FISH assay was proposed as *in vivo *biomarkers of X-radiation exposure in C57BL/6 and CBA/J mice. At the same time the comet assay alone, when applied to the same specimens, produced no significant results because of interindividual variability [59].

#### FISH in study of chromosome damage

Comet-FISH in UV-A-irradiated human lymphocytes with whole chromosome painting (wcp), centromere-, telomere- and region-specific probes demonstrated comparably high sensitivity of chromosomes X and 8 towards UV-A-induced DNA damage [60]. Studying 12 human chromosomes with wcp probes an inverse correlation between chromosomes gene density and their sensitivity towards UV-A-radiation was revealed [44].

Leukaemia-specific chromosome damage (breakage at 5q31 and 11q23) in TK6 lymphoblastoid cells exposed to melphalan, etoposide or hydroquinone was studied using Comet-FISH [61]. Comet-FISH analysis of selected genetic alterations, related with risk factors in carcinogenesis of the upper aerodigestive tract revealed significantly higher benzo[a]pyrene-diolepoxide-induced damage levels in chromosomes 3, 5 and 8 compared with chromosome 1 in epithelia cells of patients with squamous cell carcinoma [62].

**Figure 1:**
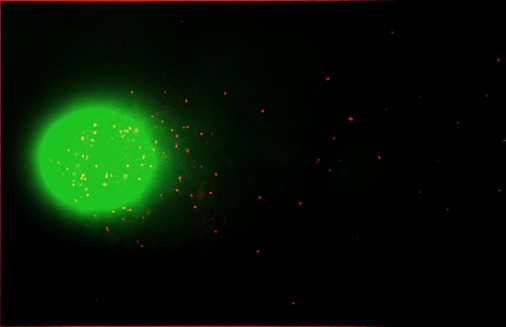
**Example of SYBR-green-stained comet image from BLM-treated human leukocytes with telomeric PNA probes indicating the location of telomeric repeat sequences**.

In our experiments Comet-FISH with telomere-specific peptide nucleic acid (PNA) probes was applied for measuring telomeric DNA sensitivity toward drugs used in cancer therapy in normal human leukocytes [63,64] and in tumor cell lines CCRF-CEM, CHO and HT1080 [65]. Distribution of telomere signals in head and tail of comet, obtained from BLM-treated human leukocytes is presented in Figure 1. Human leukocytes showed preferential cisplatin-DNA crosslinks formation in telomeres and telomere-related regions. Telomeres in CHO and CCRF-CEM cells were about 2-3 times more sensitive towards BLM than global DNA, while in HT1080 telomeres were less fragile than total DNA. The higher fragility of telomeres compared to the total DNA in non treated human leukocytes [64] reflects findings about concentration of telomeres mainly near the nuclear membrane [40].

## MN-FISH

### Methodological aspects of MN-FISH

FISH analysis of MN is based on the achievements of interphase FISH [66]. Commercial FISH probes for selective painting of individual chromosomes and specific DNA sequences and software's for image analysis are also suitable for description of MN composition. A major condition of the quantitative accuracy of the MN assay is integrity of cell membrane and preservation of the cytoplasm during the cell harvesting [67] while interphase FISH technique allows the destruction of cellular membrane.

MN test was successfully combined with different kinds of DNA probes which recognize centromeres, other chromosome-specific regions and whole chromosomes inside of micronuclei and main nuclei. The analysis of MN combined with centromeric DNA probes for all chromosomes allows discrimination between centromere negative MN or MN originating from chromosomal breakage (clastogenic effect) and centromere positive MN or MN containing whole chromosomes (aneugenic effect). Сentromere detection can be expected to be more accurate in distinguishing the two main types of MN than anti-kinetochore antibody staining [68] because MN can be formed from entire chromosomes with a disrupted kinetochore [69-71] and show no kinetochore signal.

Application of chromosome-specific centromeric probes permits evaluation of different chromosomes sensitivity toward genotoxic agents. This approach also allows detection of non-disjunctional events (i.e., unequal distribution of homologous chromosomes in daughter nuclei) in binucleated cells [72]. The application of other chromosome region-specific and wcp probes permits evaluation of their participation in formation of spontaneous [73] and induced [74,75] MN. Wcp probes target the euchromatic parts of a chromosome and thereby reveal both whole chromosomes and acentric fragments in MN [76]. However, they fail to distinguish between an entire chromosome and material from large chromosomal fragments in a particular MN. MN with whole chromosomes can be discriminated using chromosome-specific centromeric probes on the same cells [74].

However the identification of the chromosome-specific contents of MN is still very incomplete due to a lack of methods by which the DNA within the MN could be fully investigated. Moreover, the absolute number of fragments enclosed in a MN could not be quantified precisely. To our knowledge till now there are no successful attempts of application of interphase chromosome-specific multicolor banding (ICS-MCB) [27,77,78] for analysis of MN contents. Description of the chromosomal contents of MN has been limited also by the number of simultaneously applied colors and chromosomes evaluated per study. There are only a few studies with analysis of participation of all human chromosomes in MN formation. Frequency of the presence of all 24 chromosomes in MN was analyzed by dual-color FISH technique [79]. However, since only two probes on the same slide were applied, this study was time consuming. This approach was also limited in its ability to detect MN that might contain DNA from multiple chromosomes. Spectral karyotyping (SKY) technology [80] offered unique possibility for simultaneous classification of all 24 chromosomes in humans [81]. But this technology is expensive and is limited accessible in MN analysis. Wide introduction of SKY in researches provides a promising opportunity for development of our knowledge about the chromosomal contents of MN. Results of various FISH probes application with the MN test is summarized in Table 2.

**Table 2:** Results of various FISH probes application with MN test

FISH probes	What is detected	Results and applications	References
-	Both chromosome breakage and loss	Genotoxicity testing or biomonitoring of genotoxic exposure and effect	[16-26]

		Discrimination of aneugenic and clastogenic effects *in vitro*	[84-87]
		
Centromeric	MN with whole chromosomes	Discrimination of aneugenic and clastogenic effects *in vivo*	[88-92]
		
		Biomarker of radiation exposure *in vivo*	[93]

		Frequency of occurrence of chromosomes in spontaneously occurring MN	[106,107]
		
Chromosome-specific centromeric	Whole chromosomes in MN and non-disjunctional events	Comparative sensitivity of different chromosomes toward mutagens	[96,108,109]
		
		Study of mechanisms of aneuploidy	[72,94-98]

Chromosome-locus-specific	MN with chromosome loci	Nature of genome instability in tumor cells	[75]

		Distribution of radiation and chemical mutagen-induced cytogenetic damage	[74,79,105,110]
		
WCP	Both whole chromosomes and acentric fragments in MN	Composition of spontaneous and mutagen-induced MN	[39,76,79,81]
		
		Composition of spontaneous MN in cells of patients with ICF	[73]

Selected probes	Different genomic regions in MN	Elucidation of MN formation	[72,82,83]

### FISH in elucidation of MN formation

MN can arise after mitosis from acentric chromosomal fragments or whole chromosomes that are not included in each daughter nucleus. Therefore, in MN test, chromosome aberrations are detected indirectly via DNA loss from the nucleus leading to MN in the cytoplasm of the cell. FISH technique permits to identify the chromosomal origin of MN and thus improve our understanding of mechanisms of MN formation.

Anaphase aberrations and MN formation in woman lymphocytes were compared using pancentromeric and X-chromosome painting probes. It was shown, that micronucleation of the X chromosome in women's lymphocytes is probably the result of frequent lagging behind of the X chromosome during anaphase [82].

FISH with MN test permits to reveal involvement of different genes in induction of MN and aneuploidy. CBMN assay with centromere-specific probes in *XPD*-defective human fibroblast cells demonstrated that the *XPD *gene product plays a role in the events which protect human cells from the aneugenic effects of chemicals [72]. The data on the contents of MN in blood cells of workers exposed to welding fumes indicated that, detoxification gene *GSTM1 *positive subjects showed an increased centromere negative MN frequency and *GSTT1 *null subjects showed an elevated centromere positive MN frequency [83].

Thus, MN-FISH combined with analysis of anaphase aberrations and genetic polymorphisms has contributed to the understanding of the processes that accompany the formation of MN.

### MN-FISH applications in genetic toxicology

FISH analysis of MN with application of the different kinds of DNA-probes offered the possibility to precise the nature of genotoxic effects revealed in MN test. Application of MN-FISH in many researches has allowed to reveal the occurrence of different chromosomes in spontaneous and induced MN and to identify potential chromosomal targets of mutagenic substances. The clastogenic or aneugenic nature of action of many substances has been identified *in vitro *and *in vivo*. The characterization of MN contents is crucial for understanding of mechanisms of genotoxicity of chemicals and radiation.

#### Clastogenic and aneugenic effects detection and analysis of mechanisms of aneuploidy by MN test with centromeric probes

The distinction between the clastogenic and aneugenic effects (leading to structural and numerical chromosome alterations, respectively), by identifying the origin of MN, is important for genotoxicity testing or for biomonitoring of genotoxic exposure and effect. This approach with application of centromeric and chromosome-specific centromeric probes has appeared useful and widely applied in various researches.

Centromere-specific FISH analysis of the MN was applied for *in vitro *genotoxicity testing in studies of toxins of phytoplankton domoic acid (DA) and okadaic acid (OA) in human intestinal cell line Caco-2 [84], Al, Cd, Hg, and Sb salts in human blood cells [85], industrial chemical acrylamide and the traditional Chinese medicine *Tripterygium hypoglaucum *(level) hutch in mouse NIH 3T3 fibroblasts [86], anti-tumor agents cycloplatam and its parent drugs cisplatin and carboplatin in human lymphocytes [87].

MN-FISH was applied for analysis of genotoxicity *in vivo *of exposure to nitrous oxide in lymphocytes of operating-room nurses [88] and antihypertensive drug nimodipine in lymphocytes of treated patients [89]. By using FISH analysis with the mouse-satellite DNA-probe it could be shown that nicotine is a clastogen [90], while antitumor drugs topotecan and irinotecan [91] and antibiotic rifampicin [90] are aneugens as well as clastogens in somatic cells *in vivo*. The results obtained are useful for understanding of possible by-effects of action of medicines.

Environmental lead exposure increases both centromere-positive and centromere-negative MN in blood lymphocytes of children, however, the contribution of centromere-positive MN was significantly higher than in the controls [92]. The correlation between centromeric and acentromeric MN frequencies in chronically irradiated human populations and rate of exposure allows to discuss the possibility of application of centromere-specific FISH with CBMN analysis in biodosimetry [93].

Application of FISH with MN test allows not only to distinguish between clastogenic and aneugenic effects, but also enables to discriminate between two mechanisms of aneuploidy induction: chromosome loss into MN or chromosome non-disjunction so that one daughter cell becomes trisomic and the other becomes monosomic [19,72]. It was shown that chromosome migration impairment would lead to increased frequency of MN containing a single centromere whereas centrosome amplification would induce MN with three or more centromeric signals [94].

Studies with chromosome-specific centromeric probes support the observation that chromosome non-disjunction is the major mechanism of spontaneous [72,95] and induced by diethylstilboestrol [72], vincristine and demecolcine [96] and ionizing radiation [97] aneuploidy production. Chromosome loss is main mechanism of okadaic acid-induced aneuploidy [98].

#### Detection of MN contents with chromosome-specific centromeric probes

Well known fact of a non-random distribution of chromosome damages arise spontaneously [99,100] and after exposure to chemicals [101,102] or radiation [103,104] can be successfully investigated by evaluation of relative rate of micronucleation of different chromosomes or chromosome fragments [39,81,105].

With application of chromosome-specific centromeric probes in was shown that both the X and the Y chromosomes are overrepresented in lymphocyte MN of men but that the Y chromosome is overrepresented only in older subjects [106]. Occurrence of acrocentric chromosomes in spontaneous MN is neither overrepresented nor influenced by age or sex [107].

Treatment of lymphocytes with aneuploidogens vincristine and demecolcine *in vitro *increased frequency of micronucleation and malsegregation of chromosomes X and 8 in different age groups of women [96]. Aneuploidy of chromosome 8 was more frequent than aneuploidy of chromosome 7 in human lymphocytes treated with the 1,2,4-benzenetriol *in vitro *probably as only cells with non-lethal chromosome aberration could survive to be detected [108]. Non-disjunction and micronucleation of X chromosome was revealed *in vitro *in human lymphocytes treated with chemotherapeutic agents melphalan, chlorambucil and p-N,N-bis(2-chloroethyl) aminophenylacetic acid [109].

Reasons of preliminary inclusion of some chromosomes in spontaneous and induced MN require further investigation. But it is known, that chromosome-specific aneuploidy play key roles in the development and progression of cancers. Thus, precise identification of the specific chromosomes and chromosome regions involved in the observed alternations should be continuously important areas for future research.

#### Detection of MN contents with probes for chromosome regions and wcp

Analysis of chromosomal contents of spontaneous MN in normal woman lymphocytes using SKY [80] and FISH technologies demonstrated that the vast majority of MN appears to be derived from a single chromosome as a result of chromosome lagging. SKY analysis showed that all of the 23 chromosomes could be present in the MN, overall, the X chromosome was seen most frequently [81].

In spontaneously arising MN of blood cells of patients with immunodeficiency, centromeric instability, and facial anomalies syndrome (ICF) chromosome 1 appeared to be present in a higher proportion compared to chromosomes 9 and 16. Chromosome 18, not associated with the ICF syndrome, showed no signal in any of the MN observed [73]. FISH analysis of MN contents in human lymphocytes has shown lack of ethyl methanesulfonate-induced repair in chromosome 1 heterochromatin. This result is clarified frequent involvement of band 1q12 in chromosome 1 rearrangements in human cancer cells [75].

**Figure 2:**
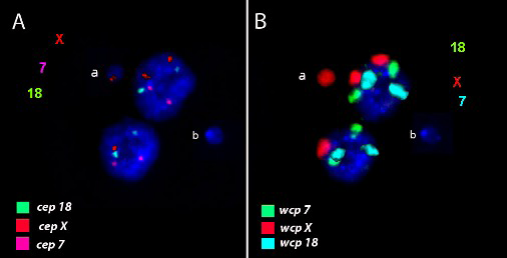
**Example of DAPI-stained binucleated cell image from MMC-treated human leukocytes with centromeric (A) and whole chromosome painting (B) probes for chromosomes 7, 18 and X**. MN (a) contains centromeric signal from chromosome X in (A) and whole chromosome probe signals from chromosome X in (B). MN (b) did not provide neither centromeric nor wcp signals.

We studied the involvement of chromosomes 7, 18 and X in mitomycin C (MMC)-induced MN using wcp and chromosome-specific centromere probes. It was shown that X-chromosomal material was over represented in female- and under represented in male-derived MN. MN with centromeric and wcp signals from chromosome X in MMC-treated human leukocytes is presented in Figure 2. We speculated about a preferred inclusion of the inactive female X chromosome into MN [74]. The contribution of different chromosomes in clastogen MMC- and aneugen diethylstilboestrol (DES)-induced MN was analysed in human lymphocytes using painting probes for all chromosomes. FISH analysis showed that DNA from chromosomes 9 and 1 was overrepresented in MMC-induced MN. The occurrence of chromosomes in DES-induced MN is more random than that in MMC-induced MN [79]. Results of application of wcp for chromosomes 1, 7, 11, 14, 17 and 21 with pancentromeric probes in MN induced by ionizing radiation in human lymphocytes support a random model of radiation-induced cytogenetic damage for the six chromosomes studied [105].

Until now, FISH has not been widely applied in plant mutagenesis because DNA probes required for chromosomes of particular plant species are very limited. The study [110] is a rare example of a detailed identification of the specific chromosomes or chromosome fragments involved in the mutagen-induced MN in barley cells.

## Conclusions

In summary, combined application of the FISH technique with comet and MN assays permits to improve the ability of these widely used genotoxicity tests.

Tests for estimation of genotoxicity belong to the express methods and should be easy and rapid in application. These advantages determine some limitations of methods, namely inability to recognize damage of certain loci of genome. MN and comet assays with application of different kinds of FISH probes offer unique possibility to detect on the same specimen the total DNA and chromosome damage and evaluate damage of specific regions of genome as well.

MN and comets appear by loss of DNA material from the nucleus in micronuclei and in comet tail, respectively. Therefore, both methods reflect secondary rather than primary effects of DNA damage. FISH analysis of origin and contents of MN and comets promotes the better understanding of mechanisms of their formation necessary for correct application of methods.

Special modifications for concurrent application of FISH with comet and MN assays were elaborated. It was confirmed that data obtained with FISH on MN and comets are comparable with results of metaphase and interphase FISH.

Comet-FISH technique permits to gain valuable and reliable information, particularly about DNA damage and repair in general and also in relation to the organization of the nucleus. Some questions relating to the behaviour and organization of DNA within the comet were clarified using FISH technique. Comet-FISH was applied for detection of DNA damage and repair of cancer relevant genes, for measuring transcription-coupled repair, identification of genome targets of action of various genotoxic agents, including anti-tumour preparations.

MN-FISH permits to discriminate aneugenic and clastogenic effect in MN and to recognize contents of MN. MN-FISH was applied in various researches for elucidation of mechanisms of genomic instability, distribution of chromosome breaks in genome and, to some extent, the etiology of certain human maladies.

Indeed, the available data demonstrate that FISH technique is able to develop the genotoxicity assessment using the comet assay and MN test.

## Competing interests

The author declares that they have no competing interests.

## Authors' contributions

GGH wrote the manuscript. Author read and approved the final manuscript.
